# Side Channel Analysis of SPECK Based on Transfer Learning

**DOI:** 10.3390/s22134671

**Published:** 2022-06-21

**Authors:** Qingqing Zhang, Hongxing Zhang, Xiaotong Cui, Xing Fang, Xingyang Wang

**Affiliations:** School of Electronic Engineering, Beijing University of Posts and Telecommunications, Beijing 100876, China; zhangqingqing24630@bupt.edu.cn (Q.Z.); cuixiaotong@bupt.edu.cn (X.C.); fancy_t@bupt.edu.cn (X.F.); wxy2020@bupt.edu.cn (X.W.)

**Keywords:** side channel analysis, SPECK, deep learning, intermediate operation, transfer learning

## Abstract

Although side-channel attacks based on deep learning are widely used in AES encryption algorithms, there is little research on lightweight algorithms. Lightweight algorithms have fewer nonlinear operations, so it is more difficult to attack successfully. Taking SPECK, a typical lightweight encryption algorithm, as an example, directly selecting the initial key as the label can only crack the first 16-bit key. In this regard, we evaluate the leakage of SPECK’s operations (modular addition, XOR, shift), and finally select the result of XOR operation as the label, and successfully recover the last 48-bit key. Usually, the divide and conquer method often used in side-channel attacks not only needs to train multiple models, but also the different bytes of the key are regarded as unrelated individuals. Through the visualization method, we found that different key bytes overlap in the position of the complete electromagnetic leakage signal. That is, when SPECK generates a round key, there is a connection between different bytes of the key. In this regard, we propose a transfer learning method for different byte keys. This method can take advantage of the similarity of key bytes, improve the performance starting-point of the model, and reduce the convergence time of the model by 50%.

## 1. Introduction

The Internet of Things revolution has led to the explosion of connected devices, such as smart cards and RFID tags and other portable devices. While traditional cryptographic algorithms such as AES are no longer suitable for these resource-limited devices, so lightweight cryptographic algorithms that meet performance and security requirements at the same time have emerged.

In 2013, NSA released the SPECK algorithm, a lightweight grouping algorithm. It can be executed on any computing device. Since its release, a lot of research has been performed on its security. Regarding SPECK algorithms resisting algebraic analytical attacks, papers [1,2,3,4] on the differential characteristics of SPECK have proven that the SPECK algorithm has good security. In terms of physical security, thesis [5,6] enhanced the ability of SPECK’s algorithm to defend against side-channel attacks by designing a microchannel anti-interference core or designing a mask, respectively.

At present, side-channel attack research based on deep learning mainly includes the following. Firstly, from the perspective of eigenvalue processing, methods such as noise reduction [7], jitter problem [8], and a class imbalance problem [9] are proposed. Secondly, from the perspective of the model construction, multi-label learning [10], integrated learning [11], multi-task learning [12], and other methods are proposed. Thirdly, from the perspective of model evaluation, a method of perfectly applying the deep learning evaluation metrics to the side-channel field is proposed [13]. Fourthly, the side-channel community has also attempted to use attentional mechanisms to enable end-to-end modeling [14] and visualization methods [15] to accurately locate informative points [16].

For the side-channel attack of AES algorithm, the attack point usually selects the nonlinear operation with large leakage such as S-box, which is helpful to distinguish the correct key from the wrong key. However, SPECK has no nonlinear operation with large leakage such as S-box, which makes it difficult to crack. In addition, when the SPECK algorithm generates the extended key, the key has “reusability”; that is, different bytes of the key are related. However, the divide and conquer algorithm ignores this problem.

In this paper, we evaluate the leakage of the existing encryption operations of the SPECK algorithm, and finally select the XOR operation as the attack point. Then, through the visual method, we analyze that the positions of information points corresponding to different bytes of the key on the leakage trace overlap, and the key “reusability” exists when the initial key generates the expansion key. Based on this relationship, we propose a transfer learning method for different byte keys.

## 2. Side-Channel Attack of SPECK

### 2.1. Specification of SPECK

**Figure 1:**
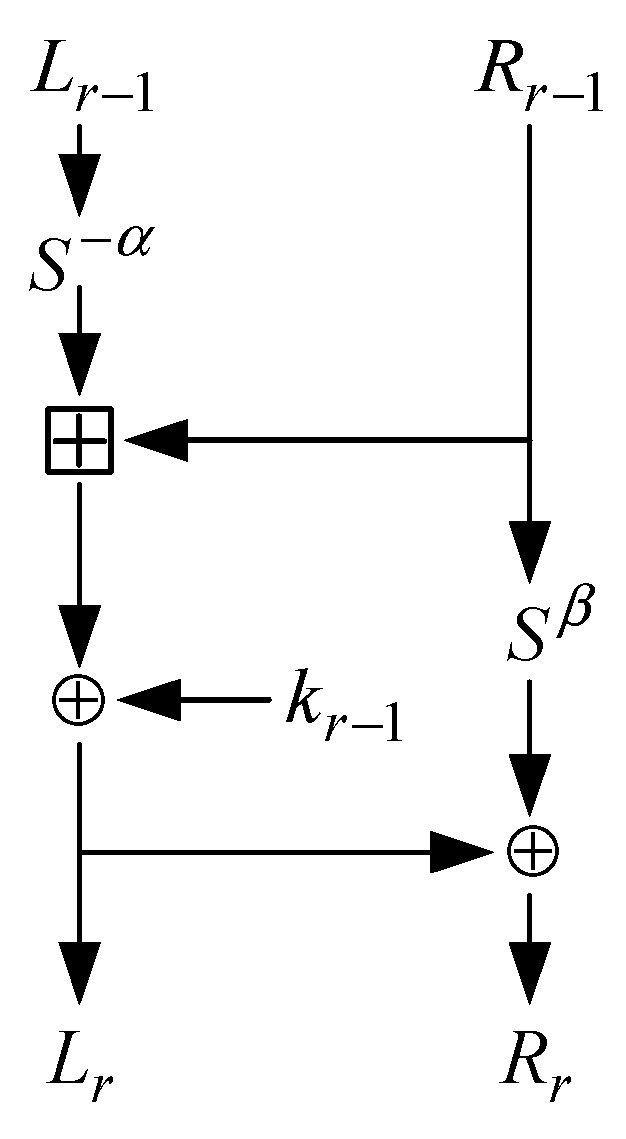
Round function of SPECK.

SPECK is a lightweight algorithm with ARX structure. The specific structure is shown in Figure 1, and its operation only involves the following three simple operations: ⊕: bitwise XOR; ⊞: an addition modulo $2^{n}$; $S^{j}$: left circular shifts by j bits, which enable high throughput and efficient implementation on resource-limited devices.

SPECK 32/64 is one of the variants of the SPECK. It has a 32-bit plaintext block and a 64-bit key block and produces a 32-bit ciphertext block as output. For an initial key, the key extension algorithm is used to generate the round key $k_{1}k_{2}k_{3},\ldots ,k_{t}$.Then, we encrypt the plaintext according to the encryption algorithm, where, r indicates that the algorithm is performing $r^{th}$ round encryption, and $k_{r}$ is the $r^{th}$ round key starting from zero.

The input at the round r−1 is represented as $\left(L_{r-1},R_{r-1}\right)$, in the same way. The output is represented as $\left(L_{r}R_{r}\right)$, the round key is represented as $k_{r-1}$, and the shift parameter is (α,β). The round function can be defined by:

(1) $$\begin{array}{l} L_{r}=(S^{-\alpha }L_{r-1}+R_{r-1})⊕k_{r-1}\\ R_{r}=(S^{-\alpha }L_{r-1}+R_{r-1})⊕(S^{\beta }R_{r-1})⊕k_{r-1} \end{array}$$

The same round function is also employed for the key scheduling, dividing the key into m 4-bit words; that is $K=(l_{m-2},\ldots ,l_{0},k_{0})$. Then, the $h^{th}$ round two sequences $k_{h}$ and $l_{h}$ are given by:

(2) $$\begin{array}{l} l_{h+m-1}=(k_{h}+S^{-\alpha }l_{h})⊕h\\ k_{h+1}=S^{\beta }k_{h}⊕l_{h+m-1} \end{array}$$

### 2.2. Attack Method

#### 2.2.1. Transfer Learning Based on Deep-Learning Method

Suppose that an attacker can access a pair of the same devices; that is, an analysis device with a known key to perform encryption operations, and a target device that performs encryption operations with an unknown key. The attacker captures the electromagnetic leakage signal from the controlled analysis device and obtains the training template, and then uses the trained template to recover the key of the target device.

The analysis of a side-channel attack based on deep learning is mainly divided into two stages: the profiling stage and the attack stage.

(1) Profiling Stage

We adopt the divide and conquer method. Taking half a byte as a division unit, we divide the n-bit key K into $\frac{n}{4}$ 4-bit words $\left(k_{1},k_{2},k_{3},\ldots ,k_{\frac{n}{4}}\right)$. On the analysis equipment, we denote $T_{profiling}=\{({\vec{l}}_{i},p_{i},k_{i})|i\leq N_{p}\}$ with $N_{p}$ labeled traces, where ${\vec{l}}_{i}$ is the $i^{th}$ collected leakage trace, $p_{i}$ and $k_{i}$ are corresponding known plaintext and known key. The intermediate value $v_{i}$ is obtained by the selection function $g(p_{i},k_{i})$, $v_{i}\in \{0,1,2,\ldots ,15\}$. Then, we build the template *Model* with the training set $T_{profiling}$. This is actually a posteriori probability model, which can give the probability that the input leakage trace belongs to all categories.

(3) $$Model=P[v_{i}|{\vec{l}}_{i}],\mathrm{where}\;v_{i}=g(p_{i},k_{i})$$

(2) Attack Stage

On the attack equipment, we denote $T_{attack}=\{({\vec{l}}_{j},p_{j})|j\leq N_{a}\}$ with $N_{a}$ leakage traces, then give a trace $T_{j}\in T_{attack}$. The attacker can compute the likelihood $d_{k_{guess_{j}}}\in R^{\left|16\right|}$ for each possible guess key $k_{guess_{j}}$ using the trained template Model. The likelihood is a vector composed of 16 prediction probabilities, and the sum of all probabilities is one.

(4) $$d_{k_{guess_{j}}}=Model(v_{j}=g(p_{j}k_{guess_{j}})|{\vec{l}}_{j})$$

If the guess key with the largest likelihood score of the sample is the correct key, the sample is successfully attacked.

(5) $$k^{*}=\underset{k_{guess_{j}}}{\arg\max}d_{k_{guess_{j}}}$$

We repeat steps 1 and 2 $\frac{n}{4}$ times until all keys are traversed. The probability of recovering the complete key is the multiplication of the probability of attacking each part of a key. This divide and conquer method reduces the search space of keys.

#### 2.2.2. Transfer Learning Based on CPA Method

The principle of correlation energy analysis attack (CPA) [17] is to crack the key by calculating the correlation coefficient between the assumed energy consumption value of the intermediate variable and the real energy signal.

The basic formula of CPA is as follows. Where $r_{i,j}$ is the correlation coefficient between the key assumption i and the sampling point j, $t_{d,j}$ is the power consumption measurement value of the $d^{th}$ energy trace at sampling point j, $h_{d,i}$ is the assumed power consumption of the $d^{th}$ energy trace when the guess key is i. There are D energy tracks. The variable $h_{d,j}$ in the equation is related to the Hamming heavy power-consumption model or the Hamming distance power-consumption model, and the higher correlation coefficient $r_{i,j}$ represents the closer guess key.

(6) $$r_{i,j}=\frac{D\sum_{d=1}^{D}h_{d,i}t_{d,j}-\sum_{d=1}^{D}h_{d,i}\sum_{d=1}^{D}t_{d,j}}{\sqrt{\left({\left(\sum_{d=1}^{D}h_{d,i}\right)}^{2}-D{\left(\sum_{d=1}^{D}h_{d,i}\right)}^{2})({\left(\sum_{d=1}^{D}t_{d,j}\right)}^{2}-D{\left(\sum_{d=1}^{D}t_{d,j}\right)}^{2}\right)}}$$

### 2.3. Leakage Model and Evaluation Metric

Intermediate value refers to the intermediate variable generated during the encryption operation of some known data (such as plaintext or ciphertext) and some unknown data (such as key byte). In order to improve the probability of successful cracking, the intermediate variable with the largest leakage is usually selected as the attack point

Guess entropy is the most commonly used index suitable for side-channel attack. However, this index is only applicable to the electromagnetic signal generated from the fixed key. The results of the index need to carry out multiple actual attacks before they are obtained, which has high time-complexity. Accuracy is the most commonly used index for monitoring and evaluating neural networks. It quantifies the true positives of all the considered positives. In addition, at the end of each epoch, the accuracy can be obtained, which is more convenient for obtaining the accuracy result. More importantly, because this paper uses the identity model as the leakage model, the category imbalance proposed in [9] has no impact on the comprehensive evaluation index. Therefore, in order to evaluate the performance of the classifier, we take the accuracy and convergence speed as an important evaluation index.

In addition, the confusion matrix can be used to evaluate the accuracy of model classification. In case of classification error, the confusion matrix can also identify the categories of confusion.

### 2.4. Visualization Method of Network Model

In order to further analyze the positions of different bytes of the key corresponding to the complete leaked signal, the trained model can be visualized. This paper adopts the visualization method Grad-CAM (Gradient-weighted Class Activation Mapping) proposed in reference [18]. This method uses the global average of the gradient to calculate the feature weight, which can reflect the attention of the network model to different positions of the leakage trajectory in the learning process. In this paper, we will scale the features of each network layer to make the output features consistent in length.

The implementation principle of Grad-CAM method is as follows. Define the weight of the $k^{th}$ feature map to the category c as $\alpha_{k}^{c}$, which can be calculated by the following formula, where z is the number of pixels of the feature map, $y^{c}$ is the score of the corresponding category c, $A_{ij}^{k}$ represents the pixel value at the (i,j) position in the $k^{th}$ feature map, after obtains the weight of the category to all feature maps, and the Grad-CAM heat-map is a weighted combination of feature maps. The reason to follow the final weighted sum by ReLU is that we only care about those pixels that have a positive impact on the category c.

(7) $$\begin{array}{l} \alpha_{k}^{c}=\frac{1}{z}\sum_{i}\sum_{j}\frac{\partial y^{c}}{\partial A_{ij}^{k}}\\ L_{Grad-CAM}^{c}=ReLU(\sum_{k}\alpha_{k}^{c}A^{k}) \end{array}$$

## 3. Experimental Analysis of SPECK Based on Deep Learning

### 3.1. Measurement Setup

**Figure 2:**
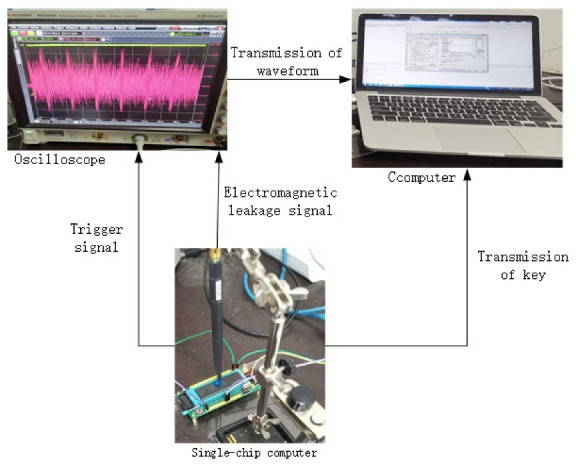
Experimental acquisition platform.

In our experiment, we implement the SPECK algorithm as MCS-51 C codes on STC12C5A60S2 (From Hongjing Technology Company, Shenzhen, China) equipped with a clock frequency of 11.0592 MHz, using a sampling rate of 50 MSa/s. The reason for choosing this single-chip computer is that the instructions are executed in sequence and the execution time is relatively short. This means that there are more encryption rounds of the leakage traces that can be collected under the condition of meeting the Nyquist sampling rate. The characteristic trace is 27,000 electromagnetic leakage traces collected by six rounds of encryption on the single-chip computer in the form of fixed plaintext and random key for a deep-learning attack. Then, while keeping the external conditions such as the single-chip computer and sampling position unchanged, we continue collected 27,000 electromagnetic leakage traces collected by four rounds of encryption on the single-chip computer in the form of random plaintext and fixed key for CPA (Correlation Power Analysis) attack. We use a trigger signal that can indicate the approximate position of the leakage trace in the encryption process. Subsequently, the original data are directly saved without any preprocessing, thus achieving end-to-end modeling. The experimental acquisition platform is shown in Figure 2.

### 3.2. Side-Channel Attack Based on Initial Key

The electromagnetic signal radiated by the chip is related to the data processed in the chip. Therefore, the electromagnetic signal radiated by the equipment executing the cryptographic algorithm contains effective information related to the key information. Electromagnetic template attack uses this feature for cryptographic analysis.

**Figure 3:**
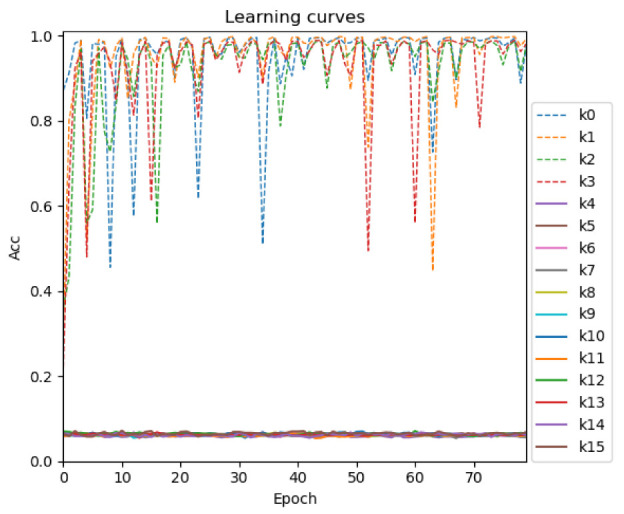
Result of directly using the initial key as the attack point to attack SPECK.

According to the attack principle of energy analysis, the most direct attack method is to directly select the initial key K as the attack target of side-channel analysis. The literature [19] proves that directly using the initial key as the attack point can successfully attack the AES algorithm. However, Figure 3 shows the result of directly using the initial key as the attack point to attack the SPECK algorithm. It is noted that for the last 48-bit key, after 80 epochs, the model still does not begin to converge. It is proven that all the keys of the SPECK encryption algorithm cannot be cracked directly using the initial key as the attack point like AES.

In order to solve this problem, we need to choose the intermediate operation with large leakage in the SPECK algorithm as the attack point to help crack the model.

### 3.3. Selection of Attack Point Location

**Figure 4:**
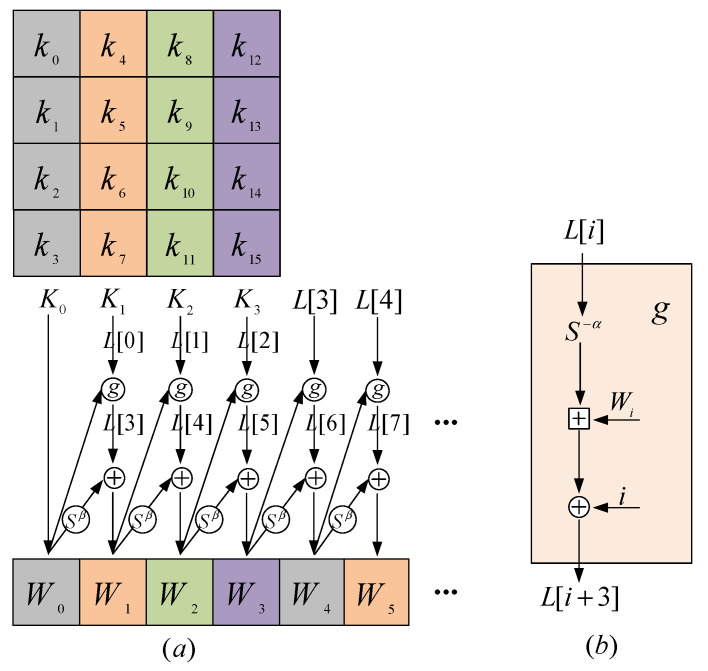
Schematic diagram of key expansion, they should be listed as: (**a**) represents the overall algorithm and (**b**) represents the function g.

It is worth noting that in the SPECK algorithm, the first 16-bit initial key is exactly the same as the first round of round key generated by the key round function, and the key is directly bound to the corresponding byte of the ciphertext, as shown in Figure 4. On the contrary, the round keys of other rounds are determined by multiple initial keys, except that the first round of encryption is directly related to the first 16-bit initial key; after that, each key expansion is related to the previous round of round key.

Therefore, we adopt the method of indirect attack. This involves how to select intermediate variables as attack points.

This may also explain why the method of directly selecting the key as the attack point can only recover the first 16-bit key information.

This paper uses the CPA (correlation power analysis) attack method and deep-learning attack method to quantify the attack efficiency of using the different encryption operation results of SPECK as the attack point. The purpose is to find the instruction with the most leakage, which helps us determine the intermediate operation with the most leakage.

By choosing to recover the $4^{th}$ half byte $k_{{}_{3}}$ of the SPECK algorithm as an example, it is not only because the carry-over problem requires leak analysis from the low-bit, but also because the high-order position bits are affected by the carry-over of the low-order position. This means that the recovery of the high-order position key is based on the completion of the recovery of the low-order position key. If the low-order byte attack fails, the subsequent bytes cannot be successfully attacked.

This paper selects the following attack points.

**The exclusive or operation:** The exclusive or operation of SPECK is an operation directly related to the key. Therefore, we choose the result of XOR between the value after modular addition and the extended key and take this result as attack point 1.

**The modular addition operation:** SPECK’s modular addition operation is the only nonlinear operation in the structure. However, each round of modular addition operation is only related to the key of the previous round, and has no direct relationship with the key of the current round. Therefore, to recover the round key of the first round, you need to select the result of the second round of module addition operation as attack point 2.

**The shift operation:** The input of SPECK’s shift operation is the encryption result of the previous round. In addition, the shift operation does not change the ciphertext, but simply changes the bit position, so this attack point is equivalent to the XOR operation.

By using CPA method and deep-learning method to attack different encryption operations of the SPECK algorithm, we can find the intermediate operation with the largest leakage and compare the attack capabilities of the two methods. Table 1 summarizes the experimental results using different attack points.

These results show that XOR operation can be used as an attack point for more effective attacks. The deep-learning method is more effective than the CPA method for cracking the key, but the deep-learning method takes more time. Therefore, it is more effective to use the CPA attack method to judge the intermediate operation with the largest leakage as the attack point.

### 3.4. Side-Channel Attack Based on Intermediate Value

As can be seen from Section 3.3, each round of XOR operation can be regarded as the attack point.

As can be seen from Figure 4, although the round function extends the key $K(K_{0}||K_{1}||K_{2}||K_{3})$ to multiple bits of the round key W, its output is fixed when the round function operates on the bytes of the key K. For example, when cracking $k_{0}$, the first half byte of the first round of round key $W_{0}$ can be selected as the attack point for side-channel attack. Meanwhile, when cracking the last 48-bit key, the XOR result of round key W and P(x) can be selected as the attack point. P(x) represents the part related to plaintext (i.e., the output of modulo addition operation). The calculation expression is as follows, in which the attacked intermediate variable φ(k) and the round key W are a one-to-one linear relationship of bits.

(8) $$W_{i}=\{\begin{array}{l} K_{i},\;\;i=0\\ g(K_{i},W_{i-1})⊕S^{\beta }W_{i-1},\;i=1,2,3,\ldots \end{array}$$

(9) $$\varphi (K_{i})=\{\begin{array}{l} W_{i},\;\;\;i=0\\ W_{i}⊕P(x),\;i=1,2,3,\ldots \end{array}$$

(10) $$\varphi (k_{i})=\{\begin{array}{l} \varphi (K_{\frac{i}{4}})>>>12,\;if\;i\;mod4=0\\ \varphi (K_{\frac{i}{4}})>>>8\&0x0F,\;if\;i\;mod4=1\\ \varphi (K_{\frac{i}{4}})>>>4\&0x0F,\;if\;i\;mod4=2\\ \varphi (K_{\frac{i}{4}})\&0x0F,\;if\;i\;mod4=3 \end{array}$$

Experimental results show that the scheme can successfully recover the last 48-bit key. At the same time, we also put forward the method of how to select the intermediate operation with large leakage.

It is worth noting that the result of each round of recovery is not the initial key K, but the intermediate value φ(k) after XOR operation. Therefore, the process of complete key recovery is to attack the output of the selection function φ(k) through the side-channel analysis, then recover the round key ($W_{0}||W_{1}||W_{2}||W_{3}$), and then solve the equations together with the given inverse function according to the key characteristics to recover the initial key ($K_{0}||K_{1}||K_{2}||K_{3}$).

**Figure 5:**
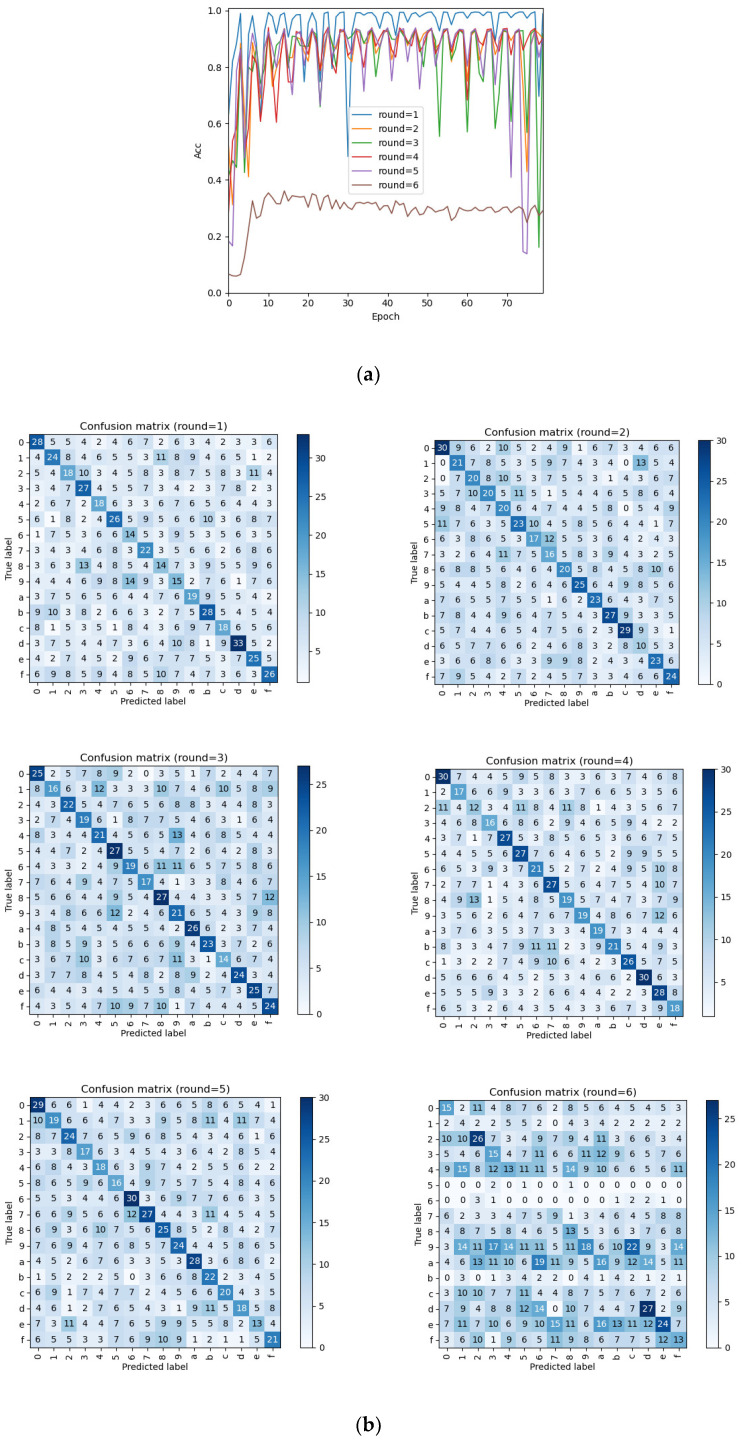
The first half byte attack result of the first six rounds of round keys; they should be listed as: (**a**) shows the learning curves, and (**b**) shows the confusion matrix.

In addition, experiments show that this method can successfully distinguish the intermediate value of the 5th round of encryption function at most. Except that the first round of round key is directly related to the initial key, the round keys of subsequent rounds are determined by multiple initial keys, and the higher the number of rounds, the richer the key information contained in the round key and the lower the attack accuracy. In addition, the attack accuracy of the high byte of the same round of round key is often higher than that of the low byte. Taking recovery of the first half byte of the first six rounds of round key $\left(k_{0},k_{4},k_{8},k_{12},w_{16},w_{20}\right)$ as an example, the attack result is shown in Figure 5.

The attack results of recovering the full key are shown in Table 2.

## 4. Side-Channel Analysis of SPECK-32/64 Based on Transfer Learning

### 4.1. Visualization of Different Convolution Layers

The visual analysis of the output position of five Conv blocks reflects the process of continuous learning and feature extraction of neural network.

**Figure 6:**
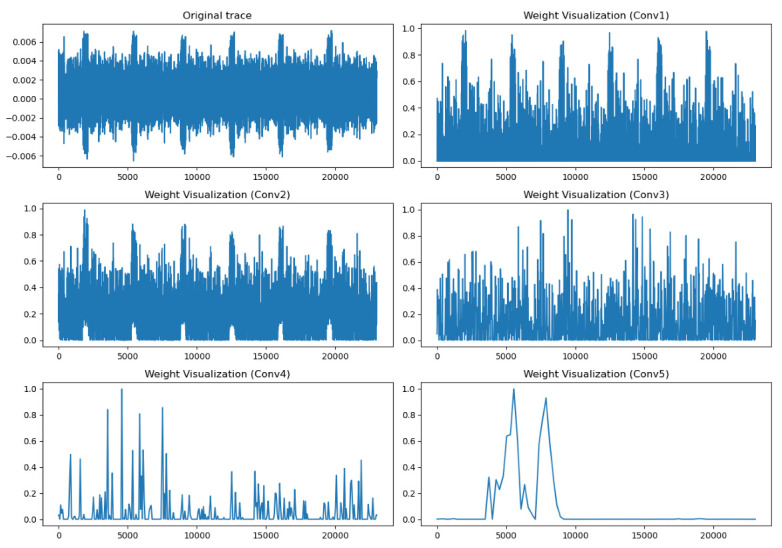
Weight visualization of different convolution layers.

Take the pretraining model constructed with the first half byte $k_{0}$ of the key K as the label as an example. Visualize the characteristic diagram of the output position of five Conv blocks, as shown in Figure 6.

Figure 6 shows that the collected leakage traces have six spikes, representing six rounds of encryption by the SPECK algorithm. We can see that the characteristic data obtained from the shallow convolution layer are still close to the original data, and six spikes can be seen, indicating that the shallow network tends to extract effective information points at the six spikes. As the number of layers is deeper, the features obtained are more and more abstract. Finally, the deep network focuses on a peak area to extract information points. It should be noted that the higher the weight, the greater the contribution of the region to the final prediction results. Through this method, the positions of different bytes of the key on the electromagnetic signal can be located.

### 4.2. Visualization of Different Key Bytes

We choose to visualize the output position of the last Conv block, which can achieve the following two purposes.

(1) It can detect the influence of the characteristic position of the leakage trace on the output results. The deeper the weight, the greater the correlation between the region and the intermediate value. It is also a useful position of information point.
(2) It can analyze the intermediate value operation and its corresponding position in the leakage trace. By study the “reusability” of key encryption and learn the correlation of different intermediate values corresponding to the position of information points on the leakage trace.

**Figure 7:**
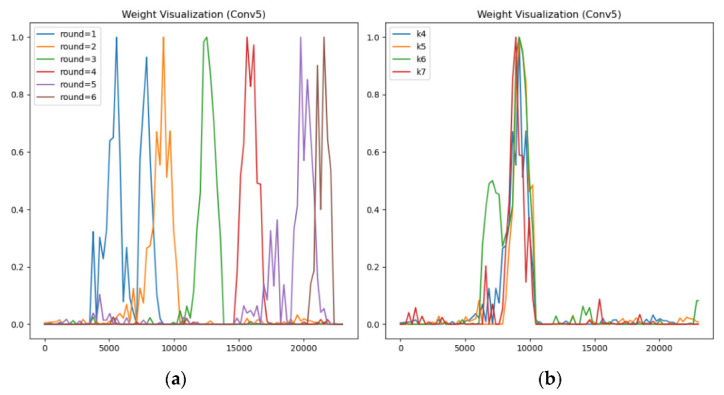
The result of weight visualization, they should be listed as: (**a**) shows the same byte position of extension keys with different rounds, and (**b**) shows the different byte position of extension key with same round.

**Experiment 1: the same byte position of round keys with different rounds:** Taking the model constructed with the first half byte $\left(k_{0},k_{4},k_{8},k_{12},w_{16},w_{20}\right)$ of the round key generated by first six rounds of encryption as the label as an example, we take the weight of the last layer of the model for visualization. It can be observed from the experiment that the positions of the information points corresponding to the six intermediate values are different, which can be roughly regarded as six peaks with intersection (as shown in Figure 7a). It proves that although these models are trained by the intermediate values obtained from different round functions, the network still extracts similar features. When generating the round key, the key not only uses the initial key corresponding to the round, but also is related to the round key of the previous round. That is, it is related to the initial key used in the previous rounds. There is the “reusability” of the key, which is reflected in the collected leakage traces and observed through visual analysis.

**Experiment 2: the different byte position of round key with same round:** Take the model constructed with the different byte positions $\left(k_{4},k_{5},k_{6},k_{7}\right)$ of the intermediate value of the second round of encryption as the label as an example, we take the weight of the last layer of these networks for visualization. From the experimental observation, the information points at different byte positions of round key with the same round are probably in the same position (as shown in Figure 7b).

Through the visualization method, we found that different key bytes overlap in the position of the complete electromagnetic leakage signal. That is, when SPECK generates a round key, there is a connection between different bytes of the key. In this regard, we propose a transfer learning method for different byte keys.

### 4.3. Side Channel Analysis of SPECK-32/64 Based on Transfer Learning

#### 4.3.1. The Reusability of Key

Through the visual analysis in the previous chapter, we find that for an algorithm such as SPECK that needs to collect multiple rounds of encrypted signals, the collected electromagnetic leakage traces will correspond to multiple rounds of complete encryption operation process. When the deep-learning method is used to execute the side-channel attack, the training model will automatically extract the information points related to the intermediate value. Although most of these information points are located in the encryption position of the intermediate value, a few are located in the encryption position of other round functions. The divide and conquer method selects some key bytes as labels and treats other key bytes as noise, which separates the correlation of different bytes of key.

According to the round function, the key has “reusability”. Except that the first round of encryption is directly related to the first 16-bit initial key, the subsequent round of encryption are participated in by multiple initial keys. The higher the number of rounds, the richer the key information. For example, the key $K_{0}$ participates not only in the first round of encryption, but also in the subsequent encryption process. Table 3 shows the key involved in first six rounds of encryption.

**Figure 8:**
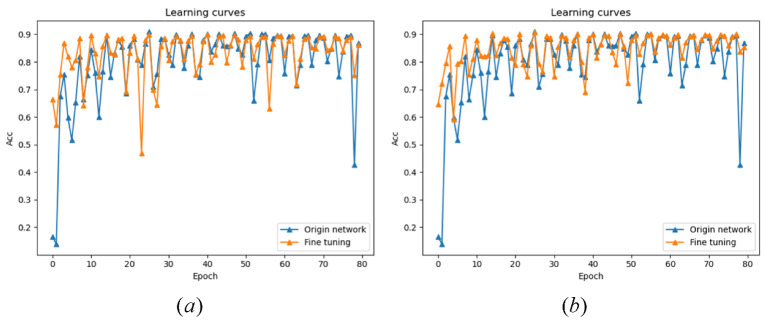
The effect of transfer learning on accuracy, they should be listed as: (**a**) shows horizontal transfer learning and (**b**) shows vertical transfer learning.

This feature is also reflected in the collected electromagnetic leakage traces. Figure 8 shows that the positions of information points extracted by different round functions have an intersection, while the information points of keys encrypted in the same round are roughly in the same position, so the features extracted by the templates constructed by these keys will be more similar. Therefore, we introduce the transfer learning method, use the fine-tuning method, take the network model trained in the source domain as the pretraining model of the target domain, and carry out training to make it adapt to the new task.

#### 4.3.2. Transfer Learning

Transfer learning can apply the knowledge or patterns learned in a certain field or task to different but related fields or problems. In the previous section, we have proven the similarity of leaked signals between different bytes of the key. Considering that most data or tasks are relevant, we can share the learned model parameters (also known as the knowledge learned by the model) to the new model in some way through migration learning, so as to speed up and optimize the learning efficiency of the model without learning from zero like most networks.

In this section, three transfer learning schemes are proposed according to the characteristics of SPECK algorithm:

**Horizontal transfer learning:** This method focuses on different bytes of the round key generated by the same round function. Visual analysis shows that the information points extracted from the template constructed by these bytes are roughly in the same position. The experiment takes the first half byte ($k_{4}$) and second half byte ($k_{5}$) of the second round of round key as the label of the source domain and the label of the target domain.

**Vertical transfer learning:** This method focuses on the same byte position of the round keys generated by different rounds. Because the key has “reusability” in the encryption process, through visual analysis, it can be seen that the information points extracted from the template constructed by these bytes have intersection. The experiment takes the second half byte of the first round of expansion key $\left(k_{1}\right)$ and the second round of expansion key $\left(k_{5}\right)$ as the label of the source domain and the label of the target domain.

The experimental results are shown in Figure 8. Through transfer learning, 65% accuracy can be achieved in the first epoch. Only 3–4 epochs can be trained to make the model converge to a better result, and the recognition accuracy is more stable. However, the training method without transfer learning needs to train at least eight epochs to achieve the same result due to the random initialization of weight, and the recognition accuracy is not stable. Therefore, the transfer learning method of side-channel attack can make use of the similarity of different bytes of the key, and has higher performance starting point, faster model convergence speed and more stable recognition accuracy.

**Different encryption operations:** In this method, we focus on the different encryption operations of the SPECK algorithm. In the experiment, the model trained by the first round of XOR results is used as the source domain, and the model trained by the second round of modular addition results is used as the target domain.

**Figure 9:**
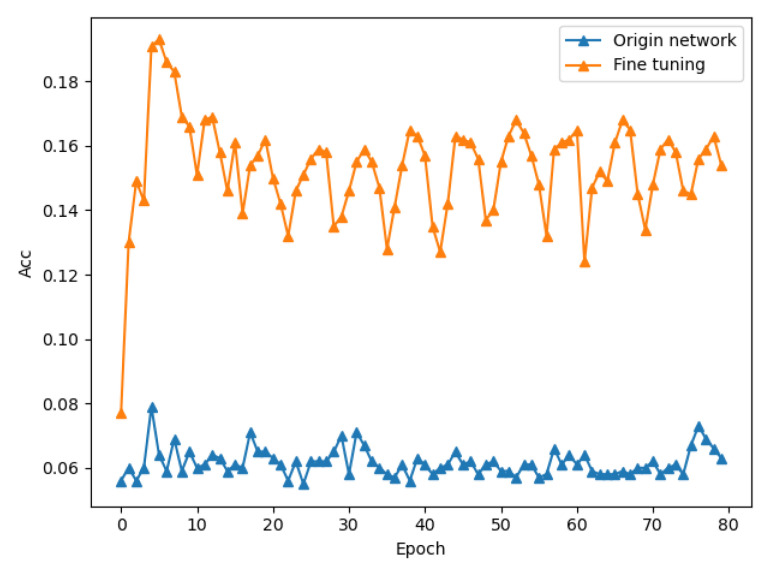
The effect of transfer learning on accuracy.

The experimental results are shown in Figure 9. Since the modulo addition operation is not an information point directly related to the key, the model training is more difficult than the XOR operation. The comparison between using transfer learning and not using transfer learning is also very obvious. Before using transfer learning, the model does not even converge, but when the XOR result is used as the pretraining model, the model begins to converge. This proves the portability of different encryption operations in the encryption algorithm.

## 5. Conclusions

### 5.1. Comparison with Other Methods

In paper [20,21], the deep-learning method is used to perform side-channel attacks. They can directly use the original key to attack the SIMECK algorithm and the AES algorithm, respectively. However, for the SPECK algorithm that needs to be broken in this paper, using the initial key as a label can only attack the first 16-bit key. To attack the complete key, we select the XOR operation as the attack point to successfully recover all keys.

Paper [6] also uses CPA method to attack the SPECK algorithm. Their experiments show that there are always interference terms that confuse the correct key when attacking modular addition. Therefore, they have to only use XOR operation as the attack point. However, the transfer learning method proposed in this paper proves that using modular addition operation as the attack point, although not as good as XOR operation, can also make the model converge. This method solves the problem of low utilization of key bytes by correlation energy analysis and powerlessness in case of key recovery failure.

We use Pearson’s correlation coefficient to evaluate the correlation between electromagnetic signals and labels. Table 4 shows the correlation coefficients of electromagnetic signals with XOR operation, initial key, and modular operation as labels, respectively. The correlation coefficient of XOR operation is the largest, the initial key is the smallest, and the modulo addition operation is the second. This is also the order of the difficulty of using these three methods as attack points.

### 5.2. Conclusions

Aiming at the problem that the lightweight algorithm is difficult to crack due to the lack of nonlinear operation with large leakage, this paper uses two attack methods of deep learning and CPA to evaluate the intermediate operation with the largest leakage, and finally selects the XOR operation as the attack point to successfully recover all keys.

Then, through visual analysis, it is analyzed that the positions of different bytes of the key on the measured signal overlap from two angles. This proves that the different bytes of the key are not unrelated individuals, and the common divide and conquer algorithm separates this relationship.

Finally, by studying the “reusability” of key encryption and using the characteristic relationship of leakage trajectory corresponding to intermediate value operation, this paper proposes a transfer learning method for different key bytes. This method can take advantage of the similarity between different key bytes, and has higher performance starting point, faster model convergence speed, and more stable recognition accuracy.

## Figures and Tables

**Table 1:** Attack results using different attack points.

Attack Method	CPA	Deep Learning
**The modular addition operation**	Attack failed	Accuracy 52.9%
**The exclusive or operation**	Attack succeeded	Accuracy 99.2%
Attack time	It takes 49 m 26 s to establish a template (attack 8-bit key)	It takes 345 m 28 s to establish a template (attack 4-bit key)

**Table 2:** The attack results of recovering the full key based on intermediate value.

Rounds	Intermediate Value	Accuracy
1	$\varphi (k_{0})$	99.6%
	$\varphi (k_{1})$	99.8%
	$\varphi (k_{2})$	98.8%
	$\varphi (k_{3})$	99.2%
2	$\varphi (k_{4})$	94.1%
	$\varphi (k_{5})$	91.0%
	$\varphi (k_{6})$	88.9%
	$\varphi (k_{7})$	85.6%
3	$\varphi (k_{8})$	93.6%
	$\varphi (k_{9})$	90.4%
	$\varphi (k_{10})$	89.2%
	$\varphi (k_{11})$	84.2%
4	$\varphi (k_{12})$	94.0%
	$\varphi (k_{13})$	89.3%
	$\varphi (k_{14})$	88.7%
	$\varphi (k_{15})$	83.5%

**Table 3:** Key involved in first six rounds of encryption.

Round	Involved Key
1	$K_{0}$
2	$K_{0},K_{1}$
3	$K_{0},K_{1},K_{2}$
4	$K_{0},K_{1},K_{2},K_{3}$
5	$K_{0},K_{1},K_{2},K_{3},K_{4}$
6	$K_{0},K_{1},K_{2},K_{3},K_{4},K_{5}$

**Table 4:** Correlation coefficients of electromagnetic signals with XOR operation, initial key, and modular operation as labels, respectively.

Correlation Coefficients	XOR Operation	Initial Key	Modular Operation
$K_{4}$	0.3072	0.0427	0.2391
$K_{5}$	0.3000	0.0269	0.1964
$K_{6}$	0.3535	0.0305	0.3274
$K_{7}$	0.2743	0.0312	0.1095

## Data Availability

The data used in this paper comes from the data measured in the laboratory.

## References

[1] Dinur I. (2014). Improved differential cryptanalysis of round-reduced SPECK. Proceedings of the International Conference on Selected Areas in Cryptography.

[2] Song L., Huang Z., Yang Q. (2016). Automatic differential analysis of ARX block ciphers with application to SPECK and LEA. Proceedings of the Australasian Conference on Information Security and Privacy.

[3] Fu K., Wang M., Guo Y., Sun S., Hu L. (2016). MILP-based automatic search algorithms for differential and linear trails for SPECK. Proceedings of the International Conference on Fast Software Encryption.

[4] Gohr A. (2019). Improving attacks on round-reduced SPECK32/64 using deep learning. Proceedings of the Annual International Cryptology Conference.

[5] Chen C., Inci M.S., Taha M., Eisenbarth T. (2016). SpecTre: A tiny side-channel resistant SPECK core for FPGAs. Proceedings of the International Conference on Smart Card Research and Advanced Applications.

[6] Ge J., Wang A., Zhu L., Liu X., Shang N., Zhang G. (2019). Power Analysis and Protection on SPECK and Its Application in IoT. Proceedings of the International Conference on Security and Privacy in Communication Systems.

[7] Wu L., Picek S. (2020). Remove some noise: On pre-processing of side-channel measurements with autoencoders. IACR Trans. Cryptogr. Hardw. Embed. Syst..

[8] Cagli E., Dumas C., Prouff E. (2017). Convolutional neural networks with data augmentation against jitter-based countermeasures. Proceedings of the International Conference on Cryptographic Hardware and Embedded Systems.

[9] Picek S., Heuser A., Jovic A., Bhasin S., Regazzoni F. (2019). The curse of class imbalance and conflicting metrics with machine learning for side-channel evaluations. IACR Trans. Cryptogr. Hardw. Embed. Syst..

[10] Maghrebi H. (2020). Deep Learning based Side-Channel Attack: A New Profiling Methodology based on Multi-Label Classification. Cryptology. ePrint Arch..

[11] Perin G., Chmielewski Ł., Picek S. (2020). Strength in numbers: Improving generalization with ensembles in machine learning-based profiled side-channel analysis. IACR Trans. Cryptogr. Hardw. Embed. Syst..

[12] Masure L., Strullu R. (2021). Side Channel Analysis against the ANSSI’s protected AES implementation on ARM. Cryptol. ePrint Arch..

[13] Zhang J., Zheng M., Nan J., Hu H., Ju N. (2020). A novel evaluation metric for deep learning-based side channel analysis and its extended application to imbalanced data. IACR Trans. Cryptogr. Hardw. Embed. Syst..

[14] Lu X., Zhang C., Cao P., Gu D., Lu H. (2021). Pay attention to raw traces: A deep learning architecture for end-to-end profiling attacks. IACR Trans. Cryptogr. Hardw. Embed. Syst..

[15] Masure L., Dumas C., Prouff E. (2019). Gradient visualization for general characterization in profiling attacks. Proceedings of the International Workshop on Constructive Side-Channel Analysis and Secure Design.

[16] Wouters L., Arribas V., Gierlichs B., Preneel B. (2020). Revisiting a Methodology for Efficient CNN Architectures in Profiling Attacks. IACR Trans. Cryptogr. Hardw. Embed. Syst..

[17] Brier E., Clavier C., Olivier F. (2004). Correlation power analysis with a leakage model. Proceedings of the International Workshop on Cryptographic Hardware and Embedded Systems.

[18] Selvaraju R.R., Cogswell M., Das A., Vedantam R., Parikh D., Batra D. Grad-cam: Visual explanations from deep networks via gradient-based localization. Proceedings of the IEEE International Conference on Computer Vision.

[19] Cui X., Zhang H., Wang L. Research on AES Cryptographic Chip Electromagnetic Attack Based on Deep Transfer Learning. Proceedings of the 2019 IEEE 6th International Symposium on Electromagnetic Compatibility (ISEMC).

[20] Wu C. (2021). Research on Side-Channel Attack of Embedded Devices Based on Machine Learning Method.

[21] Luo M., Zhang H. (2019). Research on Electromagnetic Attack of AES Cryptographic Chip Based on Deep Residual Neural Network. J. Radio Wave Sci..

